# Vitamin D modulates inflammatory response of DENV-2-infected macrophages by inhibiting the expression of inflammatory-liked miRNAs

**DOI:** 10.1080/20477724.2022.2101840

**Published:** 2022-07-19

**Authors:** Jorge Andrés Castillo, Silvio Urcuqui-Inchima

**Affiliations:** Grupo de Inmunovirología. Departamento de Microbiología y Parasitología, Facultad de Medicina, Universidad de Antioquia UdeA, Medellín, Colombia

**Keywords:** MicroRNAs, inflammation, dengue virus, vitamin D, innate immunity, IFN-I

## Abstract

Dengue disease caused by dengue virus (DENV) infection is the most common vector-borne viral disease worldwide. Currently, no treatment is available to fight dengue symptoms. We and others have demonstrated the antiviral and immunomodulatory properties of VitD3 as a possible therapy for DENV infection. MicroRNAs (miRNAs) are small non-coding RNAs responsible for the regulation of cell processes including antiviral defense. Previous transcriptomic analysis showed that VitD3 regulates the expression of genes involved in stress and immune response by inducing specific miRNAs. Here, we focus on the effects of VitD3 supplementation in the regulation of the expression of inflammatory-liked miR-182-5p, miR-130a-3p, miR125b-5p, miR146a-5p, and miR-155-5p during DENV-2 infection of monocyte-derived macrophages (MDMs). Further, we evaluated the effects of inhibition of these miRNAs in the innate immune response. Our results showed that supplementation with VitD3 differentially regulated the expression of these inflammatory miRNAs. We also observed that inhibition of miR-182-5p, miR-130a-3p, miR-125b-5p, and miR-155-5p, led to decreased production of TNF-α and TLR9 expression, while increased the expression of SOCS-1, IFN-β, and OAS1, without affecting DENV replication. By contrast, over-expression of miR-182-5p, miR-130a-3p, miR-125b-5p, and miR-155-5p significantly decreased DENV-2 infection rates and also DENV-2 replication in MDMs. Our results suggest that VitD3 immunomodulatory effects involve regulation of inflammation-linked miRNAs expression, which might play a key role in the inflammatory response during DENV infection.

## Introduction

Dengue disease caused by the transmission of the arthropod-borne dengue virus (DENV) considered a major health problem in developing countries [1]. The World Health Organization (OMS) estimates that about 50% of the world population lives in areas where the DENV transmitter mosquito *Aedes spp*. is present. Despite the high economic and social burden of dengue disease, there is no specific treatment available, and approved vaccine Denvaxia is only partially effective [2]. Furthermore, the precise mechanisms that mediate the development of severe manifestations are not fully understood. However, a number of studies have shown that uncontrolled and exacerbated inflammatory response is responsible for such complications observed in some DENV infected patients [3–5].

An exacerbated and sustained inflammatory response is a hallmark of DENV pathogenesis [3,6]. Thus, development of new therapeutics that modulate inflammatory response while also restricts DENV replication is needed. Vitamin D (VitD3) is a pleiotropic hormone that has wide immunoregulatory effect [7]. VitD3 downregulates the expression of several Toll-like receptors (TLRs) and thus contributes to the amelioration of inflammatory response triggered by interferon-gamma (IFN-γ), lipopolysaccharide (LPS), or lipoteichoic acid [8,9]. These observations suggest that VitD3 could protect from exacerbated inflammatory responses. We and others have previously shown that VitD3 decreases DENV infection and replication and the production of proinflammatory cytokines *in vitro* [10–13]. In addition, we reported a decreased susceptibility to DENV-2 infection and production of proinflammatory cytokines by monocyte-derived macrophages (MDMs) and monocyte-derived dendritic cells (MoDCs) obtained from healthy individuals who received oral supplementation of VitD3 [14,15]. This body of evidence demonstrates that VitD3 has a wide immunomodulatory and antiviral effect against DENV infection. However, specific mechanisms by which VitD3 regulates the inflammatory response during DENV infection remain unclear.

VitD3 can regulate expression of several genes that harbors Vitamin D response elements [16,17]. One of these regulated genes could encode microRNAs (miRNAs), which are small non-coding RNAs of 20–25 nucleotides that post-transcriptionally regulate the expression of a great number of genes involved in cell development, cell division, metabolism, programmed cell death, and viral pathogenesis [18]. Previous studies have shown that MoDCs treated with VitD3 produce low levels of IL-23 and show low expression of miR-155-5p, while the expression levels of miR-378 are high [19]. Similarly, VitD3 treatment of human adipocytes reduced the expression of miR-146a-5p, miR-150, and miR-155-5p during TNF-α stimulation [20]. Among these VitD3 regulated miRNAs, miR-155-5p appears to play a key role. VitD3 treatment in murine macrophages reduced the expression of miR-155-5p, which in turn increased the activity of SOCS-1 leading to a decreased inflammatory response after TLR4 activation [21]. Further, we observed that differentiation of MDMs in the presence of VitD3 (D3-MDMs) reduced miR-155-5p expression during DENV-2 infection, which was linked to increased expression of SOCS-1 [22]. Finally, we found that MDMs obtained from healthy donors who received VitD3 supplement for 10 days, showed differential expression of a set of miRNAs during DENV-2 infection *in vitro*, which target immune and cellular stress response genes [23]. The results suggest that VitD3 regulates the expression of miRNAs in inflammatory conditions.

This study aims to evaluate the modulation of inflammation-linked miRNAs by VitD3 in DENV-2-infected D3-MDMs. Further, we determined the role of these miRNAs in the inflammatory response by assessing the expression of pro-inflammatory cytokines, pattern recognition receptors (PRRs), SOCS-1, IFN-I, and IFN-stimulated genes (ISGs) in MDMs under miRNA inhibition conditions. Collectively, our results show that VitD3 treatment can modulate inflammatory response by the regulation of inflammatory-linked miRNAs in DENV-2 infected D3-MDMs, which may be associated with decreased expression of TNF-α and TLR9 and increased expression of SOCS-1, IFN-β, and OAS-1.

## Materials and methods

### Ethics statement

The protocols for sample collection were approved by the Committee of Bioethics Research of Sede de Investigación Universitaria, Universidad de Antioquia (Medellín, Colombia), and inclusion was preceded by a signed informed consent form, according to the principles expressed in the Declaration of Helsinki.

### Cells and reagents

The mosquito C6/36 HT cell line was obtained from the American Type Culture Collection (ATCC) and cultured in Leibovitz L-15 medium (Sigma Aldrich, USA) supplemented with 10% v/v heat-inactivated fetal bovine serum (FBS) (Thermo Scientific, USA), 4 mM L-glutamine, 10 U/mL penicillin, and 0.1 mg/mL streptomycin (Sigma Aldrich, USA), at 34°C in an atmosphere without CO_2_. BHK-21 cells, obtained from the ATCC, were maintained in D-MEM (Sigma Aldrich, USA), supplemented with 10% v/v FBS, 4 mM L-glutamine, 10 CFU/mL penicillin, and 0.1 mg/mL streptomycin at 37°C with 5% CO2, and used for plaque assays. A conjugated antibody against CD14 (clone M5E2) was purchased from eBioscience (USA).

### Virus stocks and titration

DENV-2 New Guinea C was provided by the Centers for Disease Control and Prevention (CDC, USA). Viral stocks were obtained by inoculating a monolayer of C6/36 HT cells in a 75-cm^2^ tissue culture flask with the virus at a multiplicity of infection (MOI) of 0.05 diluted in L-15 supplemented with 2% FBS. After 3 h of adsorption, fresh L-15 medium supplemented with 2% FBS was added, and the cells were cultured for 5 days at 34°C without CO_2_. The supernatant was obtained by centrifugation at 1000 × g for 5 min to remove cellular debris and then aliquoted and stored at −70°C until use. Virus titration was performed by quantification of plaque-forming units (PFU) using a plaque assay as described previously [13].

### Blood samples from healthy donors

Venous peripheral blood samples were obtained from healthy individuals, aged 20–40 years, who had not been previously vaccinated against yellow fever virus and were seronegative for the DENV NS1 antigen and DENV IgM/IgG, as determined by the SD BIOLINE Dengue Duo rapid test (Standard Diagnostics). All our experiments were performed with cells from at least six healthy donors.

### Monocyte isolation and monocyte-derived macrophage differentiation (MDMs)

To obtain MDMs, peripheral blood mononuclear cells (PBMCs) were obtained from 50 mL of peripheral blood from healthy individuals with 2% v/v ethylenediaminetetraacetic acid, as described previously [16,17]. Briefly, the PBMCs were separated using density gradient centrifugation and suspended in RPMI-1640 medium (Sigma Aldrich, USA) supplemented with 0.5% autologous heat-inactivated serum (30 min at 56°C). Monocytes were then obtained from the PBMCs by plastic adherence, as described previously [12]. Briefly, 5 × 10^5^ CD14+ cells into 24-well plates (Corning Incorporated Life Sciences, USA) in RPMI-1640 medium supplemented with 0.5% inactivated autologous serum and cultured at 37°C with 5% CO_2_ to allow enrichment of monocytes through plastic adherence. After 3 h, the non-adherent cells were removed by extensive washing with pre-warmed PBS supplemented with 0.5% FBS. Adherent cells were then cultured in RPMI-1640 medium supplemented with 10% FBS at 37°C with 5% CO_2_ for 6 days to obtain MDMs. Fresh medium with 10% FBS was replenished every 48 h. The purity of MDMs and D3-MDMs was repeatedly above 90%, as measured by the presence of contaminant cell populations, including CD19+, CD3+, and CD56+ in monocytes before differentiation, and measuring CD68+ cells after 6 days of differentiation.

### MDMs differentiation in the presence of VitD3 (D3-MDMs)

Monocytes were differentiated for 6 days in the presence of 1α,25-dihydroxyvitamin D3 (VitD3; Sigma Aldrich, USA), at a concentration of 0.1 nM, which represents the physiological and therapeutical concentration [23,24], as we have described previously [12,13]. The biological activity VitD3 was determined by us previously by the quantification of the transcriptional induction of VitD3 targets genes, such as VDR and CYP24A1 [12,13]. The purity of D3-MDMs was repeatedly above 90%, as measured by the presence of contaminant cell populations, including CD19+, CD3+, and CD56+ (non-myeloid cells).

### MDMs and D3-MDMs infection with DENV

Both MDMs and D3-MDMs monolayers were challenged with DENV-2 at an MOI of 5, diluted in 300 µl of RPMI-1640 medium supplemented with 2% FBS. Two hours post-infection (hpi), cells were washed with PBS, and the medium was replenished with RPMI 10% FBS and then cultured at 37°C 5% CO_2_. At 2, 8, and 24 hpi, monolayers were harvested, and either the percentage of infection was determined by flow cytometry, or total RNA extraction was carried out and used for viral RNA quantification, while the supernatants were used for viral titration by plaque assay and for quantification of cytokine production.

### Quantification of miRNA expression

Total RNA was obtained from MDMs and D3-MDMs, in both mock and DENV-2 infected samples, using the kit Direct-zol RNA miniprep (Zymo Research, USA) following the manufacturer´s instructions. The RNA concentration was quantified using a NanoDrop spectrophotometer (NanoDrop Technologies, USA). MicroRNA cDNA was synthesized from 1 μg total RNA samples using specific miRNA stem-loop primers and TaqMan MicroRNA Reverse Transcription Kit (Thermo Fisher Scientific, USA). miRNA qPCR analysis was performed in a 15 μl reaction (TaqMan™ Gene Expression Master Mix for miRNAs; Thermo Fisher Scientific, USA), and run on a Bio-Rad CFX PCR System using the following cycle conditions: 95°C for 10 mins followed by 40 cycles of 95°C for 15 secs and 60°C for 1 min. The TaqMan Assays for the following miRNAs were used: miR-182-5p (Assay ID # 000597), miR-146a-5p (Assay ID # 000468), miR-130a-3p (Assay ID # A25576), miR-125b-5p (Assay ID # 000449), miR-155-5p (Assay ID # 002623), and RNU48 (Assay ID # 001006). RNU48 was used as a reference gene to normalize the miRNA. Relative quantification of miRNA expression was evaluated using the 2-ΔΔCT method. Cutoffs for significant changes were set at p-value ≤ 0.05.

### Inhibition and overexpression of miRNAs

To inhibit the expression or overexpression assessment of selected miRNAs, MDMs were transfected with synthetic miR-182-5p, miR-130a-3p, miR-125b-5p and miR-155-5p antisense (inhibition) or mimics (over-expression) at a final concentration of 50 nM/well (Ambion, TX, USA), using DharmaFect (Thermo Scientific, NH, USA) according to manufacturer’s instructions. At 24 h post-transfection, cells were infected with DENV-2 following the procedure described above. At 24 hpi, cell monolayers were harvested and used to assess the percentage of DENV-2 infection and quantification of gene expression. The cell supernatants were used for quantification of viral RNA copies by RT-qPCR, viral titer by plaque assay, and cytokine quantification by ELISA.

### Flow cytometry assays

Flow cytometry was used to assess the frequency of DENV-infected cells [16,17]. Briefly, DENV infection was evaluated through the intracellular detection of DENV E antigen at 24 hpi fixing the cells with fixation/permeabilization buffer (eBioscience, USA). Following washing steps with PBS, cells were stained with the monoclonal antibody 4G2 (Millipore, Germany) for 40 min, followed by 40 min staining with goat anti-mouse IgG-FITC (Thermo Scientific, USA). All data acquisition and analysis were done using the BD FACScan system and FACSDiva software, respectively.

### Quantitation of viral RNA copy number

Total RNA was purified from DENV-2-infected and mock-infected MDMs using TRIzol reagent (Thermo Scientific, USA) following the manufacturer’s instructions. The RNA concentration was quantified using a NanoDrop spectrophotometer (NanoDrop Technologies, USA). Then, cDNA was synthesized using random primers from a standard concentration of 50 ng of RNA and the RevertAid H Minus First-Strand cDNA Synthesis Kit (Thermo Scientific, USA) following the manufacturer’s instructions. Viral RNA copies quantification with cDNA was carried out using specific primers, as described previously [25]. qPCR was performed with Maxima SYBR Green qPCR Master Mix (Thermo Scientific, USA) and analyzed with the CFX96 Touch Real-Time PCR Detection System (Bio-Rad, USA). The quantification of viral RNA copies was based on a standard curve of Ct values of 10-fold serial dilutions of a plasmid encoding the full genome of DENV-2 of known length and concentration, as previously described [26].

### Cytokine production

The levels of IL 6 and TNF-α were assessed in supernatants from MDMs infected with DENV-2 at 24 hpi under miRNA inhibition conditions, using an ELISA assay (BD OptEIA, BD Biosciences, USA), following the manufacturer’s recommendations.

### Quantification of gene expression

The mRNA quantification of TLR3, TLR4, TLR9, RIG-I, IFN-α, IFN-β, protein kinase R (PKR), 2ʹ-5ʹ-oligoadenylate synthetase 1 (OAS1), CAMP, SOCS-1, and ubiquitin was performed in mock and DENV-2-infected MDMs after expression inhibition of selected miRs, using qPCR. Briefly, cDNA was synthesized from total RNA using random primers, a standard concentration of 50 ng of RNA, and the RevertAid H Minus First-Strand cDNA Synthesis Kit (Thermo Scientific, USA). Then, qPCR was performed using Maxima SYBR Green (Thermo Scientific, USA), following the manufacturer’s instructions using specific primers, as described previously [25]. The specificity of the amplification product was determined by melting curve analysis. The relative quantification of each mRNA was normalized to the constitutive gene, ubiquitin, and mock-treated MDMs and D3-MDMs from each time point evaluated (e.g. 2 hours mock MDM vs 2 hours DENV-2-infected MDMs), using the ΔΔCt method and reported as the fold change.

### Statistical analysis

Comparisons between MDMs and D3-MDMs were undertaken using a two-way analysis of variance (ANOVA) along with a Bonferroni test. A value of p < 0.05 was considered statistically significant. The calculation of these parameters was carried out using GraphPad Prism version 6 (GraphPad Software, USA) software.

## Results

### VitD modulates expression of miRNAs in DENV-2 infected MDMs

Previously, we showed that a high supplement of VitD3 regulates expression of miRNAs during DENV-2 infection, which in turn regulated genes involved in stress and immune response [27]. From these differentially expressed miRNAs, we selected to study VitD3-modulated miR-182-5p, miR-130a-3p, and miR-125b-5p, because their expression has been associated with an inflammatory response in various diseases [28–30]. In addition, we included miR-146a-5p since it is overexpressed early during DENV replication, and mediates IFN-I evasion [20,31]. We also studied miR-155-5p as we and others have previously described that VitD3 can regulate its expression, and it has been associated with inflammation [21,22]. Therefore, expression of miR-182-5p, miR-130a-3p, miR-125b-5p, miR-146a-5p and miR-155-5p was quantified in MDMs and D3-MDMs during DENV-2 infection *in vitro* at 2, 8 and 24 hpi.

**Figure 1.**
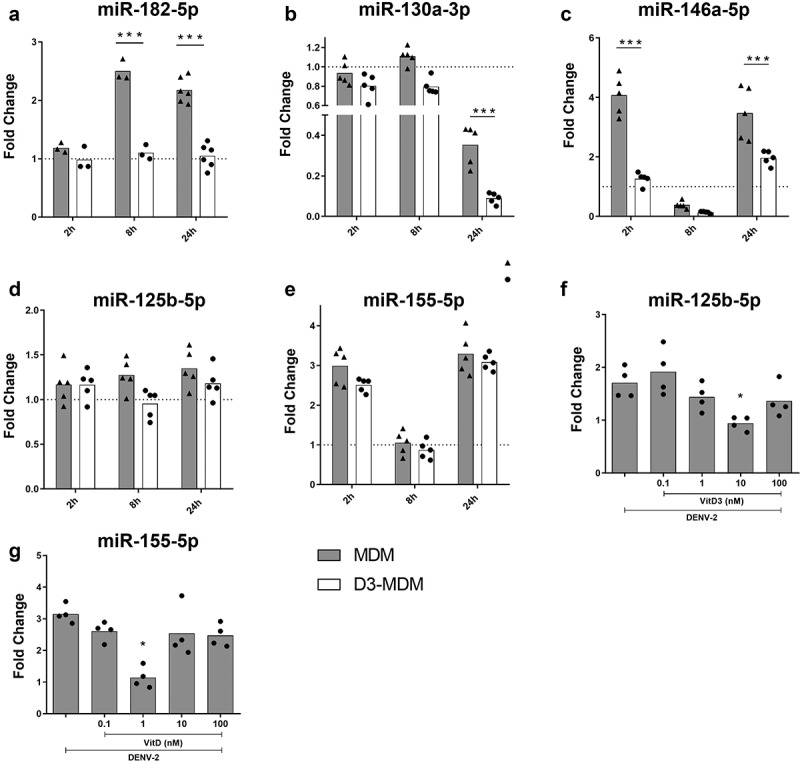
Differentiation of MDMs in the presence of VitD3 decreases the expression of miR-182-5p, miR-130a-3p, miR-146a-5p, miR-125b-5p and miR-155-5p during DENV-2 infection. The MDMs were differentiated in the presence of VitD3 (0.1 nM) for 6 days (D3-MDMs) and then infected with DENV-2 with an MOI of 5 for 2, 8, or 24 h. Expression of miR-182-5p (a), miR-130a-3p (b), miR-146a-5p (c), miR-125b-5p (d) and miR-155-5p (e) was measured by qPCR in MDMs and D3-MDMs using RNU48 as a housekeeper gene. Data are expressed as fold change relative to mock-treated MDMs and D3-MDMs from each time point. The MDMs were also differentiated with increasing concentrations of VitD3 from 0.1 to 100 nM and then infected with DENV-2 for 24 h. Expression of miR-125b-5p (f) and miR-155-5p (g) was measured by qPCR in D3-MDMs using RNU48 as a housekeeper gene. Figures represent five individual experiments. Differences were identified using a two-way ANOVA with a Bonferroni test for A-E and using a Kruskal–Wallis test for F and G. In both cases a 95% confidence interval was used (***p < 0.001, **p < 0.01, *p < 0.05).

DENV-2 induced miRNA expression in MDMs depending on the time of infection. MiR-182-5p expression increased at 8 and 24 hpi, while miR-146a-5p and miR-155-5p increased significantly at 2 and 24 hpi, compared to the mock MDMs (Figure 1a,c, and e, respectively). On the other hand, expression of miR-130a-3p in DENV-2 infected MDMs significantly decreased at 24 hpi as compared to mock-infected MDMs (Figure 1b). Notably, during DENV-2 infection of D3-MDMs, expression of miR-182-5p remained unchanged at 2, 8 and 24 hpi compared to mock-infected MDMs (Figure 1a), the expression of miR-146a-5p did not change at 2 and 24 hpi compared as well with mock-infected MDMs (Figure 1c). Similarly, expression of miR-130a-3p significantly decreased in DENV-2 infected D3-MDMs at 24 hpi as compared to both mock and DENV-2-infected MDMs (Figure 1b). We did not observe differences in the expression of miR-125b-5p and miR-155-5p between MDMs and D3-MDMs in the presence of 0.1 nM of VitD3 and in response to DENV-2 infection Figure 1(d,e). Since it has been reported that VitD3 regulates the expression of these last two miRNAs [21,32], we differentiated D3-MDMs with increasing concentrations of VitD3, followed by infection with DENV-2, and evaluated the expression of miR-125b-5p and miR-155-5p. We observed that miR-125b-5p and miR-155-5p were downregulated in D3-MDMs at VitD3 doses of 10 nM and 1 nM, respectively (Figure 1(f,g). The results suggest that VitD3 modulates expression of miR-125b-5p and miR-155-5p at different concentrations. In summary, our data indicate that VitD3 downregulates the expression of inflammatory-response miRNAs, including miR-182-5p, miR-130-3p, miR-146a-5p, miR-125b-5p, and miR-155-5p in DENV-2-infected D3-MDMs.

### Inhibition of miR-182-5p, miR-130a-3p or miR-125b-5p expression in DENV-2 infected-MDMs lead to lower production of TNF-α

VitD3 decreased the expression of miR-182-5p, miR-130-3p, miR-125b-5p, and miR-155-5p, which are shown to be associated with inflammatory response in some diseases [20,28–30]. Furthermore, we have previously shown that VitD3 can decrease the production of pro-inflammatory cytokines during DENV-2 infection of macrophages [12,13]. To test whether the decrease of miR-182-5p, miR-130a-3p, miR-125b-5p or miR-155-5p expression by VitD3 contributes to the modulation of inflammatory response of MDMs during DENV-2 infection, we transfected MDMs with anti-sense oligonucleotides against each of these miRNAs. With this strategy, a miRNA-miRNA duplex is expected to be formed inhibiting miRNA function. 24 hours after transfection, MDMs were infected with DENV-2 for an additionally 24 hours, and the production of TNF-α and IL-6 was quantified. Inhibition of each miRNA was first confirmed through qPCR. Transfection resulted in a 10-fold decrease in expression of each miRNA (Supplemental Fig 1A). Further, inhibition of these miRNAs did not affect MDMs viability under DENV-2 infection conditions (Supplemental Fig 1B).

**Figure 2.**
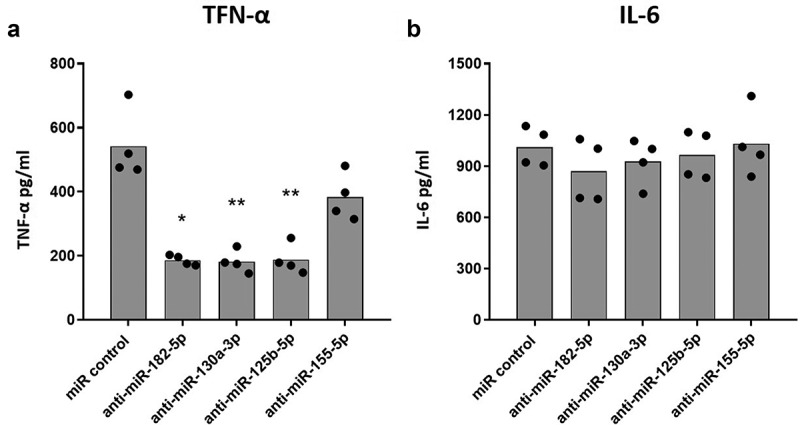
Inhibition of the expression of selected miRNAs decreases the production of TNF-α in DENV-2 infected MDMs. MDMs were transfected either with a miR scrambled (miR control) or with an anti-sense specific miRNA. 24 hours later, cells were infected with DENV-2 at an MOI of 5, and at 24 hpi, the production of TNF-α (a) and IL-6 (b) was quantified by ELISA. Figures represent four individual experiments. Differences were identified using a Kruskal–Wallis test with a 95% confidence interval was used (***p < 0.001, **p < 0.01, *p < 0.05).

While inhibition of miR-182-5p, miR-130a-3p, and miR-125b-5p significantly decreased production of TNF-α in DENV-2 infected MDMs as compared to MDMs transfected with scramble miR control (Figure 2a), inhibition of miR-155-5p did not affect TNF-α production. IL-6 production on the other hand was not altered by inhibition of the miRNAs (Figure 2b). These results suggest that miR-182-5p, miR-130a-3p, and miR-25b-5p may contribute to the inflammatory response of DENV-2 infected MDMs as they regulate TNF-α production.

### Inhibition of miRNAs expression in DENV-2 infected MDMs altered TLR9 and SOCS-1 mRNA expression

**Figure 3.**
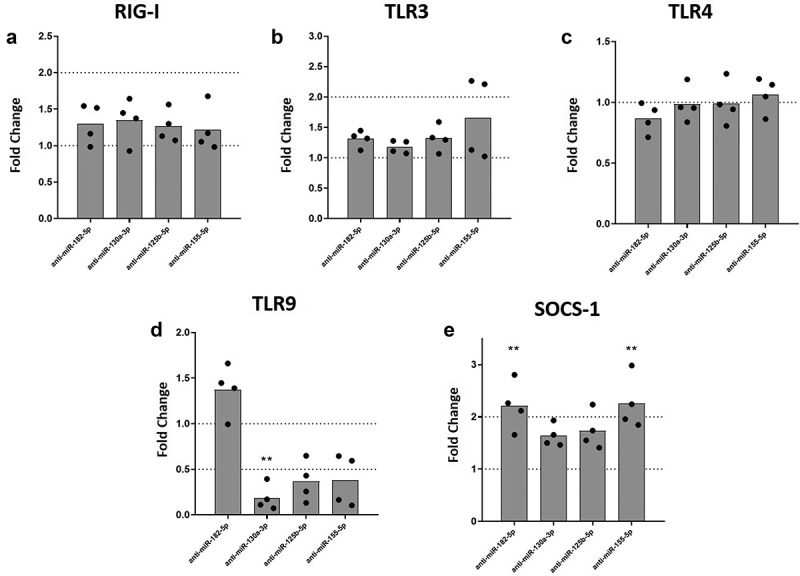
Inhibition of miR-130a-3p, miR-125b-5p and miR-155-5p leads to decreased mRNA expression of TLR9, while inhibition of miR-182-5p and miR-155-5p increases the expression of SOCS-1 mRNA in DENV-2 infected MDMs. MDMs were transfected either with a miRNA scrambled negative control or with an anti-sense specific miRNA. 24 hours later, cells were infected with DENV-2 at an MOI of 5. At 24 hpi, mRNA expression of RIG I (a), TLR3 (b), TLR4 (c), TLR9 (d), and SOCS-1 was measured by qPCR in MDMs using the gene encoding RNU48 as a housekeeper gene. Data are expressed as fold change relative to DENV-2 infected MDMs transfected with miRNA scrambled control. Figures represent four individual experiments. Differences were identified using a Kruskal–Wallis test with a 95% confidence interval was used (***p < 0.001, **p < 0.01, *p < 0.05).

Macrophages sense DENV infection through an array of PRRs including retinoic acid-inducible gene-I-like receptors (RLRs) such as RIG-I [33,34], and Toll-like receptors (TLRs) [35,36]. Activation of these PRRs can lead to the production of inflammatory cytokines. To test whether regulation of the inflammatory response was mediated by the inhibition of miRNAs and/or the regulation of PRRs, we quantified expression levels of RIG-I, TLR3, TLR4, and TLR9 mRNA under inhibition of selected miRNAs in DENV-2-infected MDMs. We found that inhibition of miR-182-5p, miR-130a-3p, miR-125b-5p or miR-155-5p, was not linked to expression of RIG-I, TLR3, or TLR4 mRNAs in DENV-2 infected MDMs (Figure 3a,b and c). However, inhibition of miR-130a-3p, miR-125b-5p or miR-155-5p decreased expression of TLR9 mRNA in DENV-2 infected MDMs, although for miR-125b-5p and miR-155-5p it was not statistically significant (Figure 3d). We further evaluated the expression of SOCS-1 under miRNAs inhibition conditions, since the protein encoded by this gene has an important role in the negative feedback of proinflammatory cytokine signaling [37]. Inhibition of miR-182-5p or miR-155-5p significantly increased the expression of SOCS-1 mRNA in DENV-2 infected MDMs as compared to infected MDMs treated with scramble control (Figure 3e). Altogether, these results suggest that inflammatory response networks involving miR-130a-3p, miR-125b-5p, and miR155-5p may be associated with TLR9 in MDMs during DENV-2 infection. Also, inhibition of miR-182-5p and miR-155-5p upregulated SOCS-1, which could contribute to the regulation of inflammatory response.

### Inhibition of miR-182-5p, miR-130a-3p, miR-125b-5p or miR-155-5p expression increased IFN-I and OAS1 mRNA levels in DENV-2 infected MDMs

**Figure 4.**
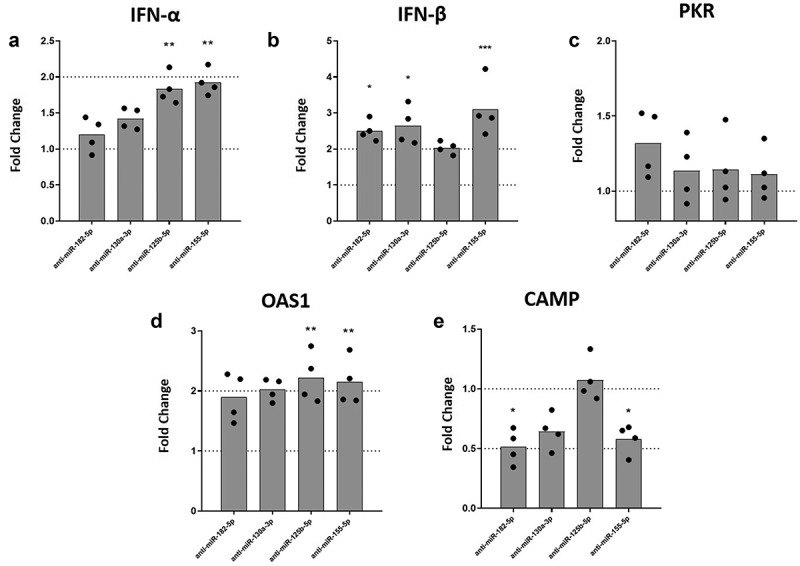
Inhibition of the expression of miRNAs differentially modulates the mRNA levels of IFN-β, OAS-1, and CAMP in DENV-2 infected MDMs. MDMs were transfected either with a miRNA scrambled negative control or with an anti-sense specific miRNA. 24 hours later, cells were infected with DENV-2 at an MOI of 5 and, at 24 hpi the mRNA expression of IFN-α (a), IFN-β (b), PKR (c), OAS1 (d) and CAMP (e) was quantified by qPCR in MDMs using the gene encoding RNU48 as a housekeeper gene. Data are expressed as fold change relative to DENV-2 infected MDMs transfected with miRNA scrambled control. Figures represent four individual experiments. Differences were identified using a Kruskal–Wallis test with a 95% confidence interval was used (***p < 0.001, **p < 0.01, *p < 0.05).

We have previously shown that VitD3 induces a partial resistance to DENV-2 infection in MDMs via down-regulation of mannose receptor [12,13]. Also, VitD3 has been shown to increase LL-37 expression [38]. To test whether inhibition of inflammatory-response inked miRNAs regulated by VitD3 could lead to an improved antiviral response, we evaluated expression of IFN-α, IFN-β, PKR, OAS1, and CAMP (LL-37 gene) under miRNAs inhibition in DENV-2 infected MDMs. Inhibition of miR-125b-5p and miR-155-5p expression induced a statistically significant increase of IFN-α mRNA levels in DENV-2 infected MDMs as compared to scrambled miRNA transfected MDMs (Figure 4a). Similarly, inhibition of miR-182-5p, miR-130a-3p, and miR-155-5p significantly increased IFN-β mRNA levels in DENV-2 infected MDMs as compared to MDMs transfected with scrambled miRNA (Figure 4b). While inhibition of miR-182-5p, miR-130a-3p, miR-125b-5p and miR-155-5p did not affect the expression of PKR mRNA (Figure 4c), inhibition of miR-125b-5p and miR-155-5p significantly increased mRNA expression of OAS1, in DENV-2 infected MDMs (Figure 4d). Surprisingly, inhibition of miR-182-5p and miR-155-5p decreased expression levels of CAMP mRNA in DENV-2 infected MDMs as compared to scrambled control (Figure 4e). Overall, the results show that inhibition of the miRNAs increases expression of IFN-I and OAS1 mRNA in DENV-2 infected MDMs.

### Inhibition of miR-182-5p, miR-130a-3p, miR-125b-5p or miR-155-5p does not alter DENV-2 replication, whereas its overexpression resulted in inhibition of DENV-2 replication in MDMs

So far, we have shown that inhibition of miR-182-5p, miR-130a-3p, and miR-125b-5p decreased the production of TNF-α, modulated the expression of TLR9, and increased expression of IFN-I and OAS-1. However, the consequence of inhibition of such miRNAs on DENV-2 replication is unknown. Therefore, we tested if inhibiting expression of miRNAs, regulated by VitD3, could also have an impact on DENV-2 replication in MDMs. For this, MDMs were transfected with anti-sense inhibitor of miR-182-5p, miR-130a-3p, miR-125b-5p, and miR-155-5p, and at 24 hours post-trasnfection (hpt), MDMs were challenged with DENV-2. Finally, at 24 hpi viral replication was evaluated.

**Figure 5.**
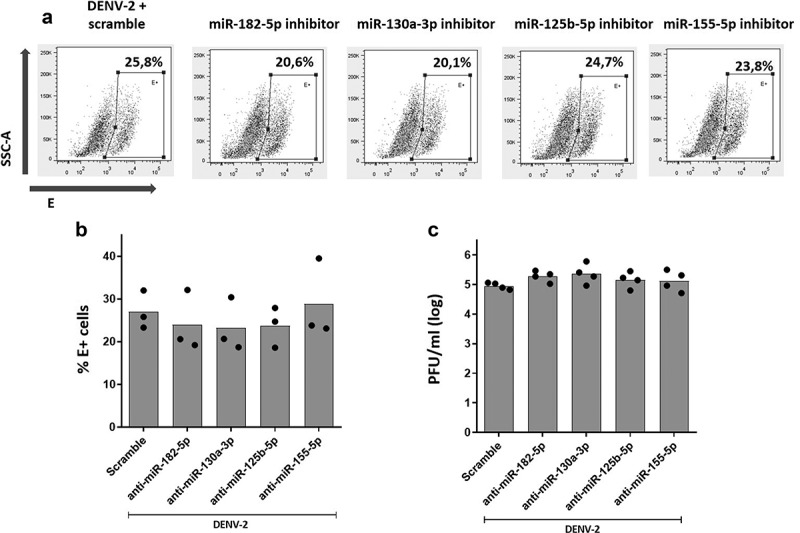
Inhibition of the expression of miR-182-5p, miR-130a-3p, miR-125b-5p and miR-155-5p did not affect DENV-2 replication in MDMs. MDMs were transfected either with a miRNA scrambled negative control or with an anti-sense specific miRNA. 24 hours later, cells were infected with DENV-2 at an MOI of 5 and, at 24 hpi, the percentage of DENV-2 infected cells was evaluated by the staining of viral envelope (E) protein and detected by flow cytometry (a, b). The infectious viral particles production was quantified at 24 hpi using plaque assay (c). Figures represent four individual experiments. Differences were identified using a Kruskal-Wallis test with a 95% confidence interval was used (***p < 0.001, **p < 0.01, *p < 0.05).

As shown in Figure 5, inhibition of miR-182-5p, miR-130a-3p, miR-126b-5p, and miR-155-5p had no effect in DENV-2 replication, since the percentage of E+ cells Figure 5(a,b) and viral titer (Figure 5c) were the same compared to control MDMs transfected with scrambled miRNA. In addition, we evaluated if overexpression of miR-182-5p, miR-130a-3p, miR-125b-5p, and miR-155-5p had any effect on DENV-2 replication. For this, MDMs were transfected with RNA mimic oligonucleotides corresponding to each miRNA, and at 24 hpt, MDMs were challenged with DENV-2. Viral replication was evaluated at 24 hpi. Overexpression of each miRNAs was confirmed through qPCR showing that transfection with miR-182-5p, miR-125b-5p, and miR-155-5p mimics resulted in a 100-fold increase, while a 50-fold increase was observed with miR-130a-3p mimic. Viability of MDMs was not affected by overexpression of miRNAs and DENV-2 infection (Supplemental Fig 2).

**Figure 6.**
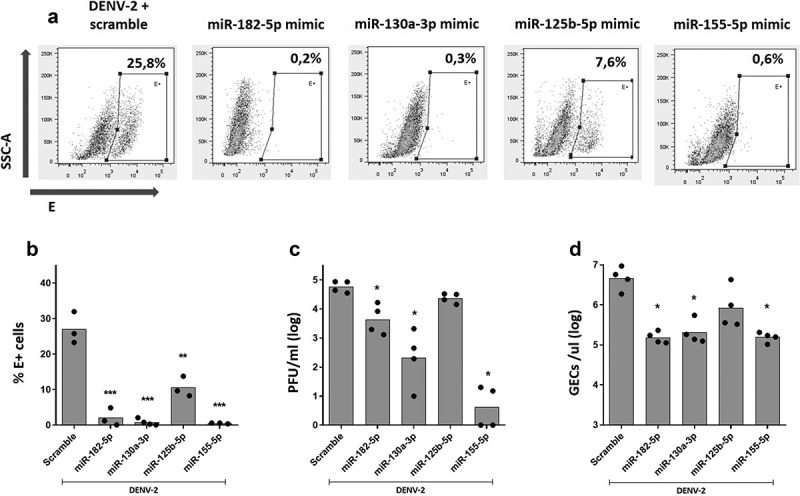
Over-expression of miR-182-5p, miR-130a-3p, and miR-155-5p inhibit DENV-2 replication in MDMs. MDMs were transfected either with a miRNA scrambled negative control or with an anti-sense specific miRNA. 24 hours later, cells were infected with DENV-2 at an MOI of 5 and, at 24 hpi, the percentage of DENV-2-infected MDMS was evaluated by the staining of the viral envelope protein (e) and detected by flow cytometry (a, b). Viral replication was evaluated at 24 hpi by the quantification of infectious viral particles using plaque assay (c) and by the quantification of the genomic equivalent copies using RT-qPCR (d). Figures represent four individual experiments. Differences were identified using a Kruskal–Wallis test with a 95% confidence interval was used (***p < 0.001, **p < 0.01, *p < 0.05).

Overexpression of miR-182-5p, miR-130a-3p, and miR-155-5p significantly decreased the proportion of DENV-2-infected MDMs Figure 6(a,b), the infectious particle production (Figure 6c), and viral RNA genome in supernatant (Figure 6d), compared to scramble miRNA transfected MDMs. Although overexpression of miR-125b-5p significantly decreased the percentage of DENV-2-infected MDMs, it did not alter the production of infectious particles or the amount of RNA genome in supernatant (Figure 6). Together, our results show that inhibition of expression of miR-182-5p, miR-130a-3p, miR-125b-5p, and miR-155-5p does not affect viral replication; however, overexpression of these miRNAs significantly decreased DENV-2 replication in MDMs.

## Discussion

MiRNAs are small non-coding RNAs that are known to regulate cellular programs including cell division, metabolism, and immune response [18]. Dysregulation of miRNA expression has been shown to impact inflammatory response (reviewed in [39,40]). A body of evidence shows that miRNAs mediate cellular responses to VitD3 [41]. In this study, we evaluated the modulation of miRNAs expression by VitD3 in MDMs during DENV-2 infection. Our data show that VitD3 differentially regulates expression of miR-182-5p, miR-130a-3p, miR-125b-5p, miR-146a-5p, and miR-155-5p, which have been associated with the inflammatory response in certain diseases [28–30] and in DENV pathogenesis [22,31]. Accordingly, inhibition of miR-182-5p, miR-130a-3p, and miR-125b-5p by antisense RNAs decreased the production of TNF-α. Due to the important role of TNF-α in DENV pathogenesis [42] and its potential to induce vascular leakage [43,44], miRNA regulation of this cytokine shed a light on the development of miRNA-based therapy.

Furthermore, we found that inhibition of miR-130a-3p expression decreased mRNA levels of TLR9 during DENV infection. This TLR has been shown to be associated with the development of inflammatory responses in certain diseases (reviewed in [45]). Also, DENV infection activates TLR9 in MoDCs leading to inflammatory response [46]. Previously, we reported that TLR9 expression is reduced in DENV-2-infected D3-MDMs [13]. These results suggest a possible link between VitD3, miR-130a-3p, and TLR9 expression. Further studies are needed to dissect the mechanism behind this link. Finally, we found an increased expression of IFN-I, OAS-1, and SOCS-1 due to inhibition of miRNA expression. Importantly, inhibition of the miRNAs did not affect DENV-2 replication, suggesting that the effect on immune response are independent of viral replication. Altogether, our results suggest that the immunomodulatory effects of VitD3 in MDMs during DENV-2 infection may be mediated by the regulation of the expression of some inflammatory-linked miRNAs.

DENV pathogenesis is mediated by uncontrolled and exacerbated inflammatory responses. Recent studies have detailed the involvement of miRNAs in the regulation of inflammation and antiviral response. For example, stimulation with LPS, Poly (I:C), CpG-ODN or IFN-β induces expression of miR-155 in murine macrophages through JNK signaling pathways [47]. Similarly, miR-125b modulates inflammatory state of macrophages by restricting the expression of B7-H4 protein, an important costimulatory molecule that suppresses T cell function [29]. Our results show that DENV-2 infection of MDMs is accompanied by increased expression of several inflammatory-response associated miRNAs, including miR-182-5p, miR-130a-3p, miR-146a-5p, miR-125b-5p, and miR-155-5p. In agreement with our results, Wang et al. demonstrated that leukotriene B4 positively regulates expression of miR-155, miR146b, and miR-125b, promoting an inflammatory state via suppression of SOCS-1 expression and increasing MyD88 expression [48]. Further, the serine/threonine kinase Akt induced by LPS stimulation in murine macrophages, upregulates the expression of miRNA let-7e, miR-155, miR-181c, and miR-125b [49]. Differential expression of inflammatory miRNAs could contribute to the development of severe dengue, since differential expression of miRNAs, including miR-6499, miR-122, miR-486, miR-383, and miR-146a, has been observed in patients with severe dengue [50–52]. Overall, miRNAs, with special emphasis on miR-155, miR146, and miR-125b, are involved in the inflammatory response of macrophages and could contribute to the exacerbated inflammation observed in DENV infection.

Innate immune response is highly regulated by the activation of transcription factor NF-κB, and its dysregulation can lead to an exacerbated/sustained inflammatory response [53]. MiRNAs have an important role in the NF-κB pathway either by regulating its activation indirectly or by being induced by NF-κB signaling [54]. Thus, inflammatory signals in hepatocytes, adipocytes, and MoDCs lead to NF-κB activation and increased expression of miR-155 and miR-146 [20,55,56]. These findings suggest that expression of these miRNAs is important for induction of inflammatory response and clearance of viral infections. Mann et al. showed a miRNA-based regulatory network in which miR-146a repressed the activation of NF-κB induced by miR-155 in mouse macrophages, representing negative feedback for the resolution of the inflammatory response [57]. In the present study, we observed increased expression of miR-146a-5p and miR-155-5p in DENV-2 infected MDMs. Surprisingly, during DENV-2 infection, the expression kinetics for these two miRNAs was the same, which may suggest that miR-146a-5p could also represent negative feedback for the inflammatory response induced by miR-155 during DENV infection. Further experiments are needed to test this hypothesis. Overall, these results suggest that miR-125b, miR-146a-5p, and miR-155 are involved in NF-κB activation and could contribute to the pathogenesis of DENV infection.

Importantly, we found that VitD3 regulated the expression of inflammatory-linked miRNAs in DENV-2-infected D3-MDMs. Since the progression to severe dengue is associated with an exacerbated inflammatory response, we propose that VitD3 regulates the innate immune during DENV infection via a miRNA-based mechanism. In other diseases with an inflammatory milieu, VitD3 has also been shown to modulate miRNAs expression. In human umbilical vein endothelial cells under treatment with serum albumin and glucose (diabetic-like environment), supplementation of VitD3 down-regulates the expression of several miRNAs [58]. Similar results have been observed in human adipocytes stimulated with TNF-α, in which VitD3 reduces the expression of miR-146a-5p, miR-150, and miR-155-5p [20]. Also, during pregnancy, there is a differential expression of miRNAs between women that show insufficient levels of circulating VitD (<25.5 ng/ml) compared to women with sufficient levels of VitD (>31.7 ng/ml) [59]. Altogether, these results show that VitD3 can regulate expression of various miRNAs involved in inflammatory disorders, including in DENV infection.

Suppressors of cytokine-signaling proteins (SOCS) are members of a family of intracellular cytokine-inducible proteins important for the regulation of inflammatory response [60]. In neutrophils treated with VitD3 and infected with *Streptococcus pneumonia*, there is increased expression of SOCS-1 and SOCS-3 compared to non-treated neutrophils [51]. We have previously shown that VitD3 treatment of MDMs increases expression of SOCS-1 during DENV-2 infection [13]. This result suggests that the upregulation of SOCS proteins by VitD3 could explain in part, its immunomodulatory activity. Here, we showed that inhibition of miR-182-5p and miR-155-5p, which are downregulated by VitD3, significantly increased the expression of SOCS-1. In agreement with our results, Chen et al. demonstrated that VitD3 treatment in mice restricted miR-155-5p expression, which in turn promoted increased expression of SOCS-1 and an attenuated inflammatory response to LPS [21]. Interestingly, SOCS-1 mRNA-3´UTR have target sequences for miR-155-5p, miR-572, miR-221, and miR-150 [61], which explains its downregulation when miR-155-5p is overexpressed. Of note, Chen et al. found that augmented expression of miR-150 and depressed expression of SOCS-1 was associated with severe dengue in patients infected with DENV [61]. Together, these results demonstrate the inflammatory potential of miR-155-5p and miR-150 during DENV infection by decreasing expression of SOCS-1, which can be further modulated by VitD3 treatment.

Of note, regulation of mRNA levels of TLR9, CAMP, IFN-I, OAS1, and SOCS-1 by miRNAs could not be confirmed at the protein levels, which is a limitation of our study. However, the main mode of action of miRNAs is mRNA decay [62], which would translate in lower quantities of mRNA as we detected by RT-qPCR. If mRNA levels correspond to protein levels should be further studied. For example, it could be that in our model, different levels of IFN-I and OAS1 mRNAs and protein are being produced, which may explain why miRNA inhibition did not affect DENV-2 replication even with higher levels of antiviral IFN-I and OAS1 mRNAs. Another possible explanation for DENV-2 replication levels under increased expression of IFN-I is the evasion of this antiviral system by DENV-2. It has been shown that DENV interferes with IFN-I signaling by blocking the activation of STAT1 and STAT2 through non-structural proteins NS4B and NS5 [63,64]. Given that STATs are required for IFN-I signaling, it could also explain why we did not observe changes in PKR mRNA expression under miRNA inhibition, even though high levels of IFN-α and IFN-β were seen. However, we could not answer why miRNA inhibition did not affect DENV-2 replication levels despite increased levels of IFN-I and OAS1, a question that could be addressed in the future.

The antiviral activity of miRNAs has been extensively described in various infections of plants, fungi, and mammals [65]. For example, miR-24 and miR-93 restrict replication of the vesicular stomatitis virus (VSV) [66], miR-29a inhibits human immunodeficiency virus 1 (HIV-1) [67], and miR-323, miR-491, and miR-654 block Influenza A H1N1 infection [68]. Surprisingly, we found that overexpression of miR-182-5p, miR-130a-3p, miR-155, and to a lesser extent miR-125b-5p, significantly inhibited DENV-2 replication in primary macrophages. Similar to our results, overexpression of miR-155 has been shown to limit DENV replication *in vitro* in Huh-7 cells and mice [69]. Further experiments revealed that miR-155 targets Bach1 resulting in inhibition of NS2B/NS3 protease activity [69]. These results highlight the key role of miR-155 during DENV infection and suggest that this miRNA could have an antiviral role during viral replication. Interestingly, Su et al. showed that miR-155 overexpression in HepG2 cells resulted in increased expression of antiviral MxA and ISG15 genes, resulting in inhibition of Hepatitis B virus (HBV) replication [70]. Our results show that DENV-2 infection upregulates expression of miR-155 suggesting that in MDMs this antiviral effect is not significant. Whether expression of miR-155 was insufficient for antiviral effect in MDMs, or whether DENV-2 actively blocks miR-155-mediated Bach1 expression requires further studies.

In addition to miR-155, we found that other miRNAs were also restricted DENV-2 replication. Although the antiviral effect of miR-182, miR-130a, or miR-125b against DENV has not been reported, other studies have shown its antiviral activity against other types of viruses. MiR-182 suppresses human cytomegalovirus by the induction of IFN-I response [71]. Over-expression of miR-130a can restrict Hepatitis C virus (HCV) replication by increasing the expression of antiviral ISG15, USP18, MxA, MX1, and OAS3 genes [72,73]. Further, miR-130a has been shown to limit HBV replication by limiting liver transcription factors PGC1α and PPARγ [74]. Similarly, overexpression of miR125b reduces replication of the flavivirus Japanese encephalitis virus (JEV) [75]. Similarly, miR-125b inhibits HIV-1 replication since its mRNA-3ʹUTR harbors seed sequences that are targeted by this miRNA [76,77]. Unfortunately, we could not dissect the mechanism of such DENV-2 inhibition. However, based on the information discussed above, we suggest that over-expression of miRNAs may enhance innate immunity-related gene expression, or indirectly, regulate the expression of key factors involved in DENV-2 replication, as we reported previously for miR-133a [78]. Altogether, these results show the antiviral potential of miRNAs against DENV infection.

In conclusion, this study demonstrated that VitD3 regulates the expression of various miRNAs involved in several inflammatory disorders, which could represent a novel immunomodulatory mechanism of VitD3 in addition to other previously reported effects, such as PRRs regulation, decrease of ROS production, and increased expression of SOCS-1 [12–14]. Our data suggest that the mechanisms behind this effect could be regulation of miRNAs expression since their inhibition had the same effect as VitD3 treatment.
